# Gut Microbiota Composition Changes following Discontinuation of Exclusive Enteral Nutrition in Children with Crohn’s Disease

**DOI:** 10.3390/microorganisms11020505

**Published:** 2023-02-17

**Authors:** Sara Sila, Marko Jelić, Ivana Trivić, Arjana Tambić Andrašević, Sanja Kolaček, Iva Hojsak

**Affiliations:** 1 Referral Centre for Pediatric Gastroenterology and Nutrition, Children’s Hospital Zagreb, 10 000 Zagreb, Croatia; 2 Division for Bacteriology, Hospital Infections and Sterilization, University Hospital for Infectious Diseases, 10 000 Zagreb, Croatia; 3 School of Medicine, University of Zagreb, 10 000 Zagreb, Croatia; 4 School of Medicine, University J.J. Strossmayer Osijek, 31 000 Osijek, Croatia

**Keywords:** microbiota, exclusive enteral nutrition, Crohn’s disease, children, adolescents

## Abstract

This study aims to determine changes in the intestinal microbiota of children with Crohn’s disease (CD) before and during exclusive enteral nutrition (EEN) and after its discontinuation. A total of 14 newly diagnosed children with CD (median age 16.0 years; 43% female) were included in this study. Patients were initially treated with EEN and were followed for one year after EEN discontinuation. Stool samples were taken at the time of diagnosis (before EEN introduction), the second day of EEN, the last day of EEN, and every two months for one year after the discontinuation of EEN. A molecular approach targeting 16S ribosomal RNA was used for analysing the gut microbiota. No change was found in the Shannon diversity index before, during, and after EEN cessation (*HhaI*-digestion *p* = 0.82; *MspI*-digestion *p* = 0.87). According to the PCO, on the basis of the dissimilarity matrices of OTUs, a clear separation of patients at different time points, forming two clusters (before and during EEN as opposed to after EEN), was evident. No clear separation was noted between patients who achieved sustained remission as opposed to those who did not achieve sustained remission during EEN and at the follow-up. In conclusion, a distinct change in the microbiota composition already occurred after two months of EEN discontinuation and remained mostly unchanged over a year of follow-up.

## 1. Introduction

Crohn’s disease (CD), a chronic, relapsing, inflammatory disorder of the gastrointestinal tract, is thought to arise from a combination of altered genetic and environmental factors acting upon the gut microbiota [1]. The exact mechanism that leads to the development of this inflammatory gastrointestinal disease has not been clarified yet. In the last two decades, special attention has been given to the role that the microbiota plays in the disease relapse and remission, especially due to successful treatment with exclusive enteral nutrition (EEN) [2,3,4,5].

EEN is used as a first-line therapy in the treatment of CD in paediatric patients [6]. The mechanism of action of EEN is not completely understood; however, multiple studies have demonstrated that EEN causes changes in the microbiota of paediatric patients with CD [7,8,9,10,11,12,13,14,15,16,17,18]. Alterations to the microbiota imposed by EEN are, therefore, assumed to be related to its effectiveness in remission induction. However, it is not clear whether the microbial shifts are caused by the EEN itself or if they are a consequence of inflammation resolution.

Although a number of studies have demonstrated changes in the microbiota composition and diversity imposed by EEN, only a few studies have examined changes that occur after the completion of EEN and a return to the regular diet [8,11,12,14,17,18]. None of the studies have a long-term follow-up of the microbiota following the termination of EEN. It is known that the effect of EEN is often transient in nature, with approximately two-third of patients relapsing within the first 12 months of EEN cessation [19,20,21,22]. In addition, recent studies exploring the impact of exclusion diets on the composition of the microbiota have demonstrated changes in gut microbiota similar to those imposed by EEN [23,24]. These studies implicate that abstention from regular food, rather than EEN formula administration, is most likely required for the effectiveness of nutritional therapy. Therefore, it is of interest to examine the microbiota changes that accompany a return to a regular diet following the cessation of EEN in CD patients.

We previously investigated the effect of EEN on microbiota composition in newly diagnosed, treatment-naïve paediatric CD patients at the following instances: before the introduction of EEN, on the second day of EEN, and at the end of EEN [7]. We found that EEN causes significant changes in the microbiota, both in CD patients and in their healthy siblings. Changes in the composition of the microbiota seem to be more uniform and occur more rapidly in healthy children. Significant changes in the direction similar to those found in healthy siblings were detected only 6 weeks after EEN introduction in patients with CD.

This study aims to assess the further effect on the microbiome after the introduction of a regular diet and every 2 months for up to one year of follow-up.

## 2. Materials and Methods

### 2.1. Patients and Study Design

Newly diagnosed paediatric patients with CD were recruited at the Referral Centre for Paediatric Gastroenterology and Nutrition at the Children’s Hospital Zagreb. In newly diagnosed CD patients for whom EEN was used as a first-line treatment, stool samples were collected at nine time points: before therapy introduction (in suspected patients, before the endoscopy was performed) (12 stool samples), on the second day of EEN (between 24 and 48 h after starting EEN) (12 stool samples), on the last day of EEN (before the introduction of regular food) (13 stool samples), and every two months for over a year after EEN discontinuation (6 samples after EEN discontinuation for each patient) (71 stool samples). Stool samples were also collected after the discontinuation of EEN and were analysed in patients who failed EEN.

In all patients, the medical chart was thoroughly reviewed and the following data were extracted: age, gender, duration of symptoms prior to diagnosis, disease location, weighted pediatric Crohn’s disease activity index (wPCDAI) at three time points (before the EEN, at the end of EEN, and a year after the discontinuation of EEN), duration of EEN, amount of EEN received per day, number of relapses in the first year from diagnosis, time (months) to first relapse during a follow-up period, partial enteral nutrition (PEN), maintenance therapy, and body mass (BM), body height (BH), and body mass index (BMI) z-scores at diagnosis and at the end of the follow-up period.

All participants older than 9 years and/or their parents provided written consent. CD was diagnosed based on the revised Porto criteria [25]. Paris classification was used to define the disease location [26]. The severity of the disease was estimated using the wPCDAI [27]. EEN failure was defined as the inability to normalize inflammatory markers and reduce symptoms during the course of EEN, in addition to the need to step up to corticosteroid therapy. Based on the wPCDAI, remission was defined as <12.5 points, mild disease activity was defined as 12.5–40 points, moderate disease activity was defined as 41–57.5 points, and severe disease activity was defined as >57.5 points [10]. Furthermore, to consider patients as in remission, calprotectin levels should have been lower than 150 ug/mg. Sustained remission was defined for those patients who achieved remission during EEN and had no relapse in the first 3 months after EEN cessation.

EEN was administered for 6 to 8 weeks. The choice of formula depended on the age and taste preference of the patient (five patients received Osmolite^®^, Abbott; four patients received Pediasure^®^, Abbott; three patients received Ensure Plus^®^, Abbott; one patient received Resource Junior^®^, Nestle; and one patient received Modulen IBD^®^, Nestle). After EEN cessation, all patients received the same advice from the dietitian regarding the implementation of the Mediterranean diet and the avoidance of highly processed foods.

Stool samples were stored at −20 °C for a maximum of 24 h, after which they were transferred in cold packs to the Department of Clinical Microbiology at the University Hospital for Infectious Diseases and stored at −80 °C.

### 2.2. Microbiota Analysis

A molecular approach targeting 16S ribosomal RNA was used for analysing the gut microbiota. Microbiota analysis procedures were performed using PCR amplification and terminal restriction fragment length polymorphism (T-RFLP), previously described in detail in Sila et al. (2021) [7].

### 2.3. Statistics

Differences between the samples were analysed by ANOVA; a post hoc analysis was performed using the Tukey test (with a Bonferroni adjustment, in which *p*-values were multiplied by the number of tests being carried out). A related-samples Friedman’s two-way analysis of variance by ranks was used to compare the wPCDAI values for each patient at three time points and to compare the Shannon–Wiener diversity index at nine time points. A related-samples Wilcoxon signed rank test was used to compare the BW, BH and BMI z-scores at diagnosis and at the end of follow-up for each patient.

The relative abundance of operational taxonomic units (OTUs) was used to calculate the Shannon–Wiener diversity index with an aim to compare diversity between different sample groups. BioNumerics software (Applied Maths, Sint-Martens-Latem, Belgium) was used to perform cluster analyses based on the *HhaI* or *MspI* T-RFLP patterns. Due to a high number of variables in the OTU comparisons, *p* values were adjusted for multiple comparisons (*p* value was decreased by 5 times). Values below 0.01 were considered significant.

A permutational multivariate analysis of variance (PERMANOVA) (fixed effect, using the type III sum of squares and the unrestricted permutation of data with 999 permutations) was used to analyse the difference in abundance of OTUs between patients and sample times. Data were transformed (square root), and the Bray–Curtis measure was used to assess dissimilarities. Centroids were determined for every sample time point (baseline, on day two, on the last day of EEN, and every two months post-EEN for one year). A principal coordinates ordination analysis (PCO) was used to visually present the dissimilarities between patients and sampling time (nine sample times).

Statistical analysis was performed using SPSS 23.0 (Chicago, IL, USA) and Primer 7 software (Auckland, New Zealand).

The study was approved by the Ethics Committee of the Children’s Hospital Zagreb (IRB number: 21102014).

## 3. Results

A total of 14 patients with CD patients were included in this study. The general data of all included patients are shown in Table 1. Two patients (14.3%) failed EEN. The other 12 (85.7%) patients achieved clinical remission, defined by wPCDAI scores ≤12.5, by week eight. By month three after EEN cessation, ten patients (71.4%) remained in remission and were classified as sustained remission (SR). Three (21.4%) patients, who were malnourished and did not achieve their energy requirements from the diet alone, received partial enteral nutrition (PEN), supplying an additional 200 kcal to one patient, 400 to 600 kcal to a second patient, and 600 to 900 kcal to a third patient.

A significant decrease in wPCDAI was found from diagnosis to the end of EEN (median 42.5 (32.5, 110) at diagnosis vs. median 5 (0, 75) at the end of EEN; *p* < 0.001) and after one year of follow-up (median 42.5 (32.5, 110) vs. median 0 (0, 42.5); *p* < 0.001).

A significant increase in the BW z-score (median at diagnosis −0.0 (−2.0, 1.3) vs. median at the follow-up 0.3 (−1.4, 2.1), *p* = 0.016) and BMI z-score (median at diagnosis 0.1 (−3.4, 1.2) vs. median at the follow-up 0.3 (−2.0, 2.0), *p* = 0.03) was detected when comparing said measures at diagnosis to the measures at the follow-up.

**Figure 1:**
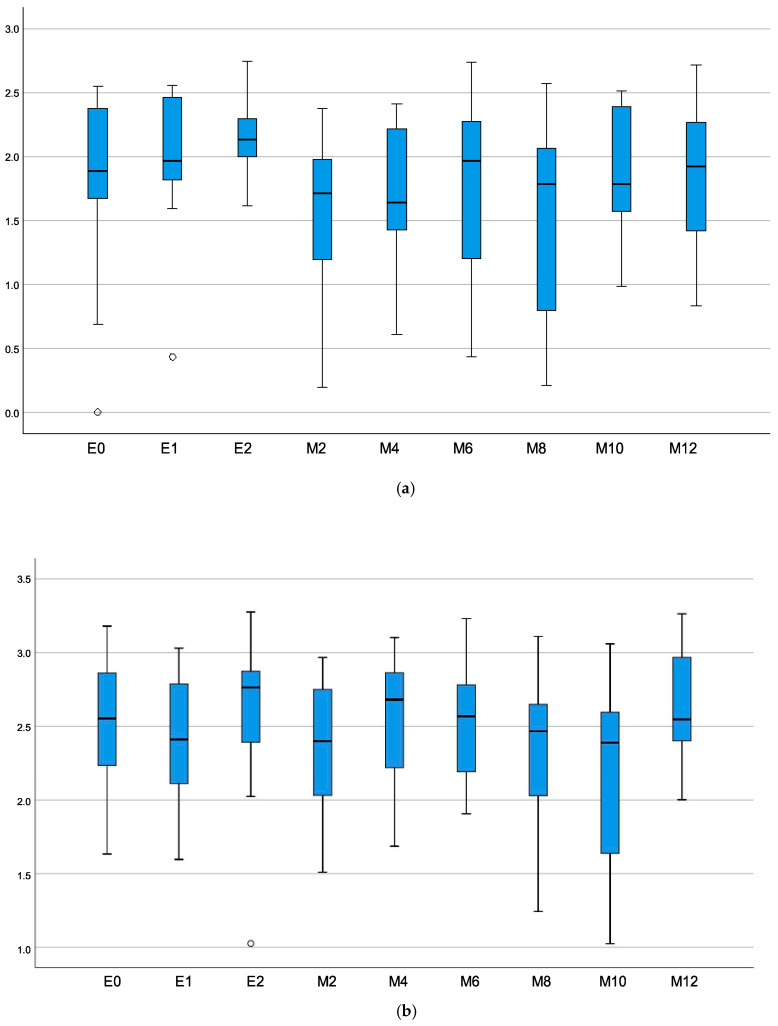
Shannon diversity index for each sample time for (**a**) *HhaI*-digestion and (**b**) *MspI*-digestion. There was no difference in microbiota diversity in CD patients at the diagnosis and while on EEN (second and last day of EEN and during follow-up).

The change in the diversity of the microbiota (Shannon index) is shown in Figure 1. The Shannon diversity index did not change significantly before, during, and after EEN cessation (*HhaI*-digestion *p* = 0.82; *MspI*-digestion *p* = 0.87).

E0—patients at baseline; E1—patients on day 2 of EEN; E2—patients at the end of EEN; M2—2 months after the cessation of EEN; M4—4 months after the cessation of EEN; M6—6 months after the cessation of EEN; M8—8 months after the cessation of EEN; M10—10 months after the cessation of EEN; and M12—12 months after the cessation of EEN.

Significant differences in the abundance of bacteria were observed for 3 out of 189 OTUs (1.6%; *HhaI*-digestion) and 8 out of 583 OTUs (1.4%; *MspI*-digestion). A post-hoc analysis revealed those differences were related to the significant decrease from diagnosis to the second day of EEN (85-bp *MspI* OTU, 96-bp *MspI* OTU, 281-bp *MspI* OTU, 489-bp *MspI* OTU), a significant increase on the second day of EEN (75-bp *HhaI* OTU, 130-bp *HhaI* OTU, 117-bp *MspI* OTU, 490-bp *MspI* OTU), or the significant increase on the last day of EEN (740-bp *HhaI* OTU, 101-bp *MspI* OTU). For one OTU (486-bp *MspI* OUT), an increase that started from the sixth month continued until the last follow-up. The increase in abundance of bacteria is represented by 138-bp *MspI* OTU (*Bacillus, Eubacterium, Paenibacillus, Desulfotomaculum*), 148-bp *MspI* OTU (*Bacillus, Clostridium, Paenibacillus, Staphylococcus*), 149-bp *MspI* OTU (*Bacillus, Desulfovibrio, Firmicutes, Paenibacillus*), 172-bp *MspI* OTU (*Collinsella, Desulfotomaculum, Bacteroides*), 281-bp *MspI* OTU (*Catenibacterium, Bacillus, Fusobacterium, Propionibacterium, Ruminoccocus, Klebsiella, Escherichia*), and 554-bp *MspI* OTU (*Streptococcus, Enterococcus, Lactococcus*). There were also 718-bp *MspI* OTU (non-specified) detected between the sixth and eighth months of follow-up; however, the difference was not significant. Nevertheless, for the OTUs described above, a high abundance was detected at the start of EEN (at the diagnosis and/or the second day of EEN), followed by a drop in abundance at the end of EEN therapy and a rise in abundance at the sixth to eighth month post-EEN, which was similar to that detected at the start of EEN.

The PERMANOVA displayed a significant difference in the microbial communities for *HhaI*-digestion between patients at different time points (df = 15, MS = 3666.1, pseudo F = 2.0824, *p* = 0.001) and sampling times (df = 8, MS = 3631.4, pseudo F = 2.0627, *p* = 0.001), and for *MspI*–digestion for patients (df = 8, MS = 4803.3, pseudo F = 1.5816, *p* = 0.001) and sampling times (df = 14, MS = 5894.5, pseudo F = 1.9409, *p* = 0.001).

**Figure 2:**
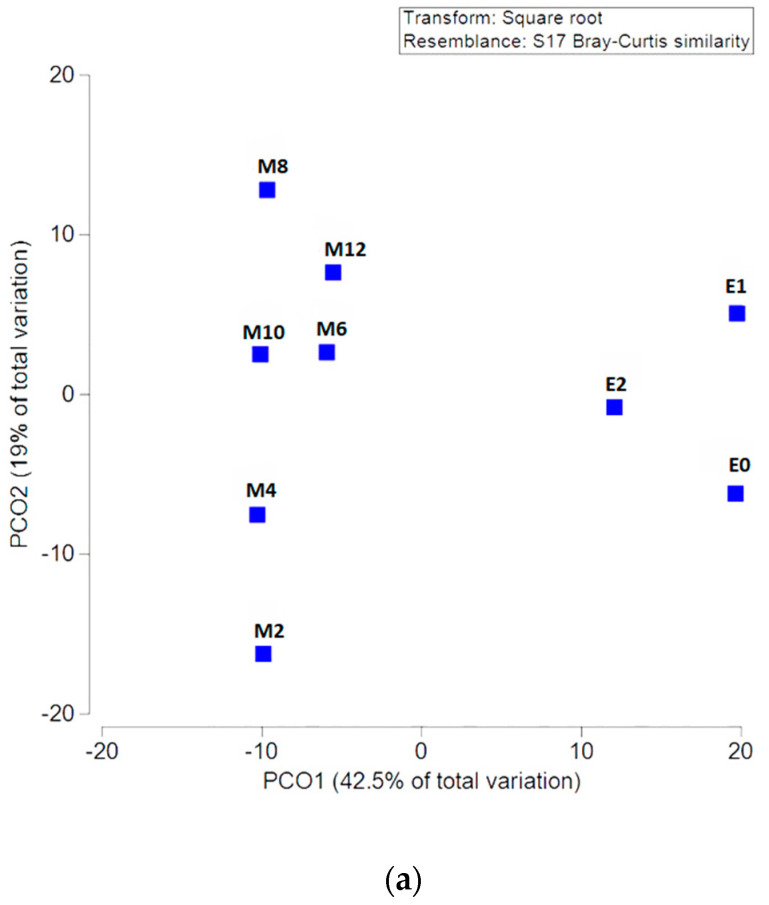

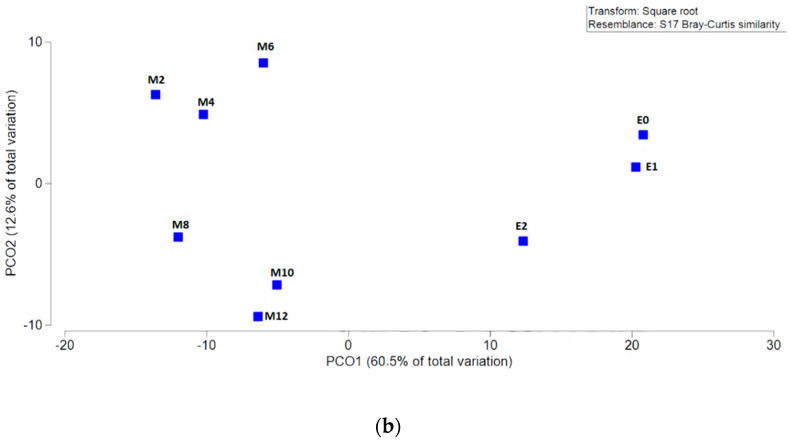
Principal coordinate ordination analysis (PCO) based on the OTU for (**a**) *MspI*-digestion, and (**b**) *HhaI*-digestion showing different time points of EEN and post-EEN treatment. PCO was analysed using Bray-–Curtis distances with centroids (3b).

**Figure 3:**
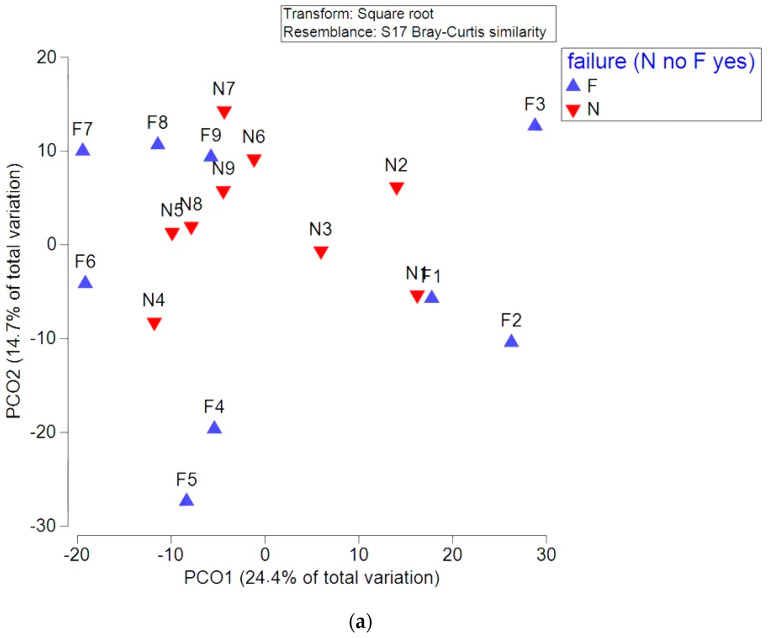

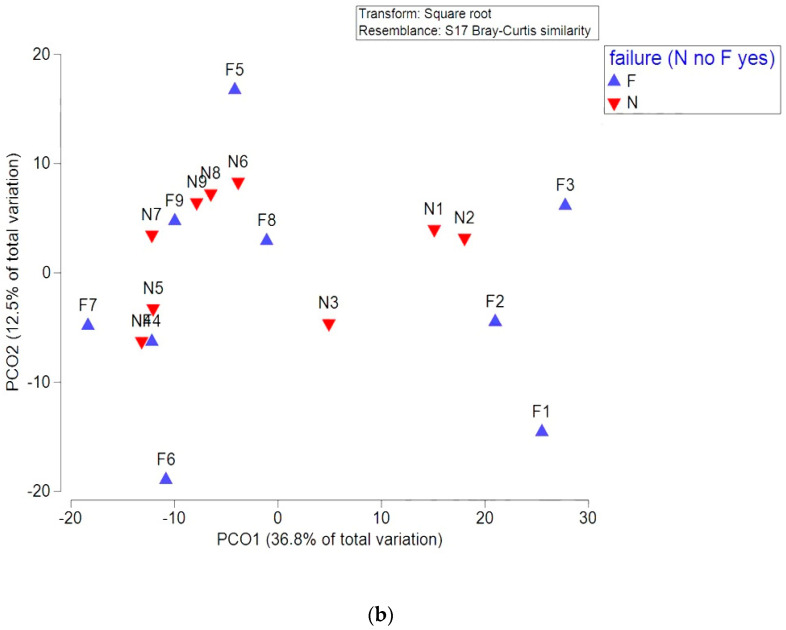
Principal coordinate ordination analysis (PCO) based on the OTU for (**a**) *MspI*-digestion, and (**b**) *HhaI-*-digestion showing two groups of patients (sustained remission (N) vs. non-sustained remission (F)) at different time points of EEN and post-EEN treatment. PCO was analysed using Bray–Curtis distances with centroids (3b).

Figure 2 and Figure 3 represent the PCO on the basis of dissimilarity matrices of OTUs for *HhaI*-digestion and *MspI*-digestion; there is a separation between patients at different time points into two clusters (before and during EEN as opposed to after EEN).

E0—patients at baseline; E1—patients on day 2 of EEN; E2—patients at the end of EEN; M2—2 months after the cessation of EEN; M4—4 months after the cessation of EEN; M6—6 months after the cessation of EEN; M8—8 months after the cessation of EEN; M10—10 months after the cessation of EEN; and M12—12 months after the cessation of EEN.

N—sustained remission; F—non-sustained remission.
N1/F1—patients at baseline; N2/F2—patients on day 2 of EEN; N3/F3—patients at the end of EEN; N4/F4—2 months after the cessation of EEN; and N5/F5—4 months after the cessation of EEN.

According to the PCO, there was no clear separation on the basis of dissimilarity matrices of the OTUs was observed for patients who achieved sustained remission as opposed to those who did not achieve sustained remission (Figure 3) during EEN and at the follow-up. Both groups of patients showed similar shifts in microbiota composition.

## 4. Discussion

In this follow-up study of CD patients treated with EEN, we assessed changes in microbial communities following a return to a free diet over a one-year period. A PERMANOVA showed changes in the microbial communities that followed EEN treatment, with a major shift already occurring 2 months after EEN cessation. Further shifts in the microbiome over a one-year period of follow-up were milder and showed clustering and clear separation from the period during which EEN was used. Importantly, microbial communities did not return to the pre-treatment levels as shown, pointing to a sustained modulation of the microbiota composition occurring after EEN treatment. However, this could equally be the result of the successful treatment and the decrease in inflammation.

We previously showed that significant changes in microbial communities already occur on the second day of EEN and continue until the last day of EEN [7]. That effect was also described by others [2,5], yet only a limited number of studies have performed a follow-up after a return to the free diet, and most commonly only at one time point [8,11,12,14,17,18]. By employing a PERMANOVA, we have demonstrated that the most significant shift in microbiota communities occurs after EEN cessation, 2 months post-EEN. After that time point, slight shifts are still detectable over a one-year period, but at no time point does the microbiota composition return to its pre-treatment levels (Figure 2). This observation was also made by Leach et al. [14] at the fourth month follow-up. Two other studies [8,18], however, demonstrated that the overall microbial composition at the follow-up was not different from the microbiota composition observed before the introduction of EEN, indicating that the microbiota reverted to its pre-treatment levels on a free diet.

Interestingly, according to the PCO, on the basis of the dissimilarity matrices of OTUs, (Figure 3) we did not detect differences in the microbiota composition of patients who achieved a sustained remission after EEN as opposed to those who failed EEN or who did not achieve sustained remission. However, step-up therapy with corticosteroids or biologics was used in those patients, and all of them ultimately achieved remission. As shown by Hart et al. [28], the gut microbiota of paediatric CD patients treated with either EEN or corticosteroid therapy was largely independent of the choice of treatment but was mostly influenced by patients’ success or failure to achieve remission. Since the majority of our patients ultimately achieved remission, no difference in failures vs. non-failures was detected. This again suggests that the reduction in gut mucosal inflammation might be the main contributing factor for a sustained change in microbiota composition.

Although most changes in the microbial composition (as detected by abundances of specific OTU-s) were detected during EEN (on the second day and the last day of EEN), shifts were also observed during the sixth and eigth months post-EEN. More specifically, an initial decrease at the start of EEN followed by a rebound at the sixth and the eighth month post-EEN was detected for OTUs predicting bacteria from the genera *Staphylococcus, Firmicutes, Ruminococcus, Desulfotomaculum, Desulfovibrio, Collinsella, Klebsiella, Escherichia, Enterococcus, Streptococcus, Bacillus, Paenibacillus*, and *Clostridium*. Potentially, changes in microbiota composition that occur at this time could cause a disruption of microbiota that leads to inflammation and, consequently, a relapse of the disease. Still, the relapse of the disease itself could pave the way to changes in microbiota composition that we detected. Additionally, according to our data, it seems that the overall microbiota composition was not changed at the sixth to the eighth month post-EEN according to the PCO on the basis of dissimilarity matrices of OTUs (Figure 2 and Figure 3).

A number of studies have shown a significant decrease in the diversity of the microbiota during EEN [5], a finding that we did not confirm. Similarly, a recent study by Jones et al. [17] did not observe changes in the diversity of the microbiota during the course of EEN. Additionally, no change in microbial diversity was observed during the one-year period of follow-up.

To our knowledge, this is the first study that followed microbiota composition for one year after the cessation of EEN and during the return to a free diet. Nevertheless, this study has some limitations: primarily, a small number of subjects. Moreover, by using a molecular approach targeting 16s ribosomal RNA gene, we were not able to detect strain-level taxonomic classification. While the nutritional intake during EEN was uniform in all patients, returning to the free diet can have a divergent effect on the microbiota composition of CD patients, especially since a proportion of these patients continued receiving partial enteral nutrition in addition to a free diet. These variables can certainly have a profound impact on the microbiota composition; however, we were not able to take these into consideration. Moreover, maintenance therapy and the use of corticosteroids could have affected the microbiota of these patients; however due to a small study sample, we were not able to statistically analyse the impact of different maintenance therapies and corticosteroids on the microbiota composition of these patients. Nevertheless, the main aim of our study, which was to detect if, and in what way, microbial communities continue to change after EEN, was reached.

## 5. Conclusions

In general, there is already a distinct change in the composition of the microbiome of children with CD after 2 months of the discontinuation of EEN. This effect remains unchanged during a one-year period of follow-up. Importantly, our study did not show that the microbiota composition returns to the composition observed before the introduction of EEN, indicating a sustained modulation after EEN treatment in these patients.

## Figures and Tables

**Table 1:** Demographic data of patients with Crohn’s disease at diagnosis (*n* = 14).

Patients with Crohn’s Disease (*n* = 14)	
Age (years), median (minimum, maximum)	16.0 (13.2, 18.0)
Gender, female, *n* (%)	6 (42.9%)
Duration of symptoms before diagnosis (months), median (minimum, maximum)	3 (0, 9)
Disease location, *n* (%)	
L1	5 (35.7%)
L2	1 (7.1%)
L3	8 (57.1%)
Upper gastrointestinal disease, *n* (%)	7 (50.0%)
wPCDAI, median (minimum, maximum)	42.5 (32.5, 110)
Maintenance therapy, *n* (%)	
Azathioprine	11 (78.6%)
Methotrexate	1 (7.1%)
None	2 (14.3%)
BW z-score, median (minimum, maximum)	−0.0 (−2.0, 1.3)
BH z-score, median (minimum, maximum)	−0.1 (−1.2, 2.0)
BMI z-score, median (minimum, maximum)	0.1 (−3.4, 1.2)
Duration of EEN (weeks), median (minimum, maximum)	6 (2, 8)
Daily amount of EEN formula, median (minimum, maximum)	1900 (1500, 2400)
Partial enteral nutrition in the first year after diagnosis, *n* (%)	3 (21.4%)
Number of relapses in the first year from diagnosis (in EEN non-failures), median (minimum, maximum)	0 (0, 4)
Time to the first relapse (months) (in EEN non-failures), median (minimum, maximum)	2 (0, 6)

BH—body height; BMI—body mass index; BW—body weight; EEN—exclusive enteral nutrition; wPCDAI—weighted pediatric Crohn’s disease activity index.

## Data Availability

Not applicable.
